# The Stroke Hyperglycemia Insulin Network Effort (SHINE) trial: an adaptive trial design case study

**DOI:** 10.1186/s13063-015-0574-8

**Published:** 2015-03-04

**Authors:** Jason T Connor, Kristine R Broglio, Valerie Durkalski, William J Meurer, Karen C Johnston

**Affiliations:** Berry Consultants, LLC, 4301 Westbank Dr Bldg B Suite 140, Austin, TX 78746 USA; University of Central Florida College of Medicine, 6850 Lake Nona Blvd, Orlando, FL 32827 USA; Department of Public Health Sciences, Medical University of South Carolina, 135 Cannon Street Suit 303, Charleston, SC 29425 USA; Department of Emergency Medicine, University of Michigan Health System, 1500 E Medical Center Dr, Ann Arbor, MI 48109 USA; Department of Neurology, University of Virginia Health System, PO Box 800394, Charlottesville, VA 22908 USA

**Keywords:** Bayesian adaptive design, Group sequential, Sample size estimation, Predictive probabilities, Neurologic emergencies

## Abstract

**Background:**

The ‘Adaptive Designs Accelerating Promising Trials into Treatments (ADAPT-IT)’ project is a collaborative effort supported by the National Institutes of Health (NIH) and United States Food & Drug Administration (FDA) to explore how adaptive clinical trial design might improve the evaluation of drugs and medical devices. ADAPT-IT uses the National Institute of Neurologic Disorders & Stroke-supported Neurological Emergencies Treatment Trials (NETT) network as a ‘laboratory’ in which to study the development of adaptive clinical trial designs in the confirmatory setting. The Stroke Hyperglycemia Insulin Network Effort (SHINE) trial was selected for funding by the NIH-NINDS at the start of ADAPT-IT and is currently an ongoing phase III trial of tight glucose control in hyperglycemic acute ischemic stroke patients. Within ADAPT-IT, a Bayesian adaptive Goldilocks trial design alternative was developed.

**Methods:**

The SHINE design includes response adaptive randomization, a sample size re-estimation, and monitoring for early efficacy and futility according to a group sequential design. The Goldilocks design includes more frequent monitoring for predicted success or futility and a longitudinal model of the primary endpoint. Both trial designs were simulated and compared in terms of their mean sample size and power across a range of treatment effects and success rates for the control group.

**Results:**

As simulated, the SHINE design tends to have slightly higher power and the Goldilocks design has a lower mean sample size. Both designs were tuned to have approximately 80% power to detect a difference of 25% versus 32% between control and treatment, respectively. In this scenario, mean sample sizes are 1,114 and 979 for the SHINE and Goldilocks designs, respectively.

**Conclusions:**

Two designs were brought forward, and both were evaluated, revised, and improved based on the input of all parties involved in the ADAPT-IT process. However, the SHINE investigators were tasked with choosing only a single design to implement and ultimately elected not to implement the Goldilocks design. The Goldilocks design will be retrospectively executed upon completion of SHINE to later compare the designs based on their use of patient resources, time, and conclusions in a real world setting.

**Trial registration:**

ClinicalTrials.gov NCT01369069 June 2011.

## Background

In 2010, The National Institutes of Health (NIH) and the Food and Drug Administration (FDA) jointly awarded four grants to support research in the area of regulatory science [1]. One of these awards, ‘Adaptive Designs Accelerating Promising Trials into Treatments (ADAPT-IT)’, is a collaborative effort between the University of Michigan, Medical University of South Carolina, and Berry Consultants, LLC. The overall objective of ADAPT-IT is to explore how adaptive designs in a confirmatory setting might improve the evaluation of drugs and medical devices in neurologic emergency settings [2]. ADAPT-IT uses the NINDS-supported Neurological Emergencies Treatment Trials (NETT) network as a ‘laboratory’ in which to study the development of adaptive clinical trial designs. The NETT Network includes 22 hubs, along with a clinical coordinating center (University of Michigan), and statistical and data management center (Medical University of South Carolina) and focuses on conducting large trials in acute injuries and illnesses affecting the brain, spinal cord, and peripheral nervous system [3]. For each of five randomized clinical trials undergoing proposal development for implementation within NETT, the ADAPT-IT investigators developed an adaptive trial design in collaboration with each study’s principal investigators and statisticians at the data coordinating center. During this process another research team used both qualitative and quantitative methods to characterize the beliefs, opinions, and concerns of the trial’s key stakeholders regarding the ethics, scientific validity, and integrity of the adaptive designs and how these beliefs may have changed over the course of the design process.

The ADAPT-IT process begins with an initial meeting of clinical investigators and statisticians to describe the scientific question and sketch out potential designs. An initial adaptive design is then constructed and presented to the trial team for feedback. There are several iterations of the design and accompanying discussions. Once a final design is agreed upon, the trial is submitted for funding.

Each of the five trials included in ADAPT-IT were at varying stages of development and funding. The first trial considered within ADAPT-IT, the Stroke Hyperglycemia Insulin Network Effort (SHINE) trial had a completed design. Also, unlike the other four trials, SHINE was approved for funding by the NIH-NINDS at the time the ADAPT-IT project began. Regardless, through the ADAPT-IT process, an alternative adaptive design was developed. We present the SHINE trial design that is currently being conducted, which is an adaptive design including group sequential stopping, response adaptive randomization, and a blinded sample size re-estimation. We also present the alternative ADAPT-IT design, which is a Bayesian adaptive Goldilocks trial design [4]. We compare the performance of the two designs via simulation and then describe how SHINE will be virtually re-executed according to the Goldilocks design.

There are examples where an alternative trial design has been retrospectively created and executed in order to compare the potential benefits of different design features [5,6]. However, SHINE is a unique learning opportunity in that the same team developed, in a prospective manner, two different trial designs with the same scientific objectives in mind. In the trial design process, it is typical to consider several different candidate designs. One design is selected for conduct and the alternatives are discarded. However, we propose to consider both the selected design and the counterfactual, comparing and contrasting both the design and the execution of two innovative adaptive trial designs.

The two designs we will describe have several differences, and therefore, this is not an ‘apples to apples’ comparison where we compare one simple difference at a time. However, this is also not an academic exercise in which the goal is to consider only the difference between the frequentist and the Bayesian perspectives. Rather, we present a more ‘real world’ setting in which two candidate designs are weighed against each other based on their own unique sets of benefits and drawbacks.

## Methods

### SHINE trial design

The SHINE trial design is completely described elsewhere [7]. SHINE is a randomized multicenter Phase III trial comparing tight glucose control with IV insulin (experimental) to a therapy of subcutaneous insulin (control) in hyperglycemic acute ischemic stroke patients. The SHINE protocol was approved by IRBs at the University of Virginia, the NETT Clinical Coordinating Center (University of Michigan), and Statistics and Data Management Center (Medical University of South Carolina), as well as all enrolling sites. The name of the ethical body that approved the SHINE protocol at each enrolling site is shown in the appendix. Consent is obtained either from patients, or where cognitively impaired from stroke, a legally authorized representative, before a patient is enrolled in the SHINE trial.

The primary efficacy endpoint is a dichotomized modified Rankin scale (mRS), adjusted to the baseline stroke severity score (NIHSS), measured at 90 days following randomization. The null hypothesis is that the success rates for the two arms are equal. The alterative hypothesis is two-sided, that the success rates for the two arms are not equal. The alternative hypothesis will be accepted if either the experimental therapy is significantly better than the control or if the control therapy is significantly better than the experimental. Based on preliminary data, it is expected that 25% of patients in the control group will achieve success. At least a 7% absolute difference in the proportion of patients achieving a success between the treatment and control arms would be considered clinically meaningful. Based on a chi-square test, four interim analyses, an overall 0.05 Type I error rate, a control success rate of 25%, and a 3% lost to follow-up rate, 1,400 patients would provide 80% power to detect the 7% absolute difference between treatment arms.

SHINE will be monitored for both early efficacy and futility according to a group sequential design. The stopping boundaries are defined by the gamma family spending function with a parameter of −4 for both the upper and lower bounds. Four interim analyses are scheduled when complete information on the primary endpoint (90-day data) is available for 500, 700, 900, and 1,100 patients. The two-sided *P* values required for stopping for efficacy or futility at each of these interim looks are shown in Table 1. To control the overall two-sided Type I error rate to less than 5%, the final analysis will be conducted at the 0.043 significance level. The primary efficacy analysis will be a logistic regression model with terms for treatment group, baseline NIHSS strata, and use of IV thrombolysis use (yes or no). Multiple imputation will be implemented when the 90-day outcome is missing or collected outside of the allowable window of −14 day/+30 days from the 90-day visit.

**Table 1:** **SHINE two-sided** ***P*** **values for stopping for success or futility at each interim analysis**

**Information fraction**	**Success** ***P*** **Value**	**Futility** ***P*** **Value**
36%	0.003	0.949
50%	0.004	0.896
64%	0.008	0.652
78%	0.016	0.293
100%	0.043	0.043

SHINE also contains a sample-size re-estimation analysis to ensure 80% power if the control success rate is higher than expected and variance is increased. The sample size re-estimation will follow the approach of Gould and Shih [8], and will be based on the observed overall success rate and assuming a 7% absolute difference between treatment arms. If the overall pooled success rate is greater than 31%, the maximum sample size may be increased. As specified in Gould and Shih, to maintain the blind, the sample size re-estimation is planned just prior to the first unblinded interim analysis. If the sample size is increased, the timing of the interim looks will be adjusted accordingly to preserve the planned information fraction at each look. The largest possible increase to the maximum sample size would be 318 patients.

Finally, patients will be randomized to either the experimental or control arm based on a randomization scheme that includes both covariate balancing and response adaptive randomization. Covariates to be balanced are the baseline NIHSS (3 strata), use of IV thrombolysis (yes or no), and site. As SHINE is currently ongoing, to prevent operational bias, the details of the response adaptive randomization component of the design have not been provided to the SHINE study investigators who are potentially enrolling patients and are not included in this manuscript.

### Goldilocks design alternative

The only constraints applied to the alternative design for the SHINE design were having a maximum sample size of 1,400 patients, and the assumptions of a control rate favorable outcome of 25% where a 7% improvement would be clinically meaningful. All other design parameters were subject to change.

The alternative SHINE trial design is a Bayesian adaptive Goldilocks design that includes frequent interim looks, based on predictive probabilities, to stop early for efficacy or futility [4,9]. Because patients can also be assessed for mRS at 6 weeks, we use a longitudinal model of the primary endpoint to allow the 6-week measure of mRS to aid prediction of mRS at 90 days. Patients are equally randomized to either the experimental or control arm. The minimum sample size is 500 patients, and the maximum sample size is 1,400 patients and there are scheduled interim analyses after every additional 100 patients are enrolled. The primary analysis is fully Bayesian, comparing the posterior distributions of the success rates between the two arms. In this design, the alternative hypothesis is one-sided, that the success rate on the experimental arm is greater than on the control arm. The rationale for a one-sided alternative hypothesis is that the clinical implications of either futility (the two treatments being similar) or the experimental therapy being worse than control are the same - patients would continue to receive the control treatment, the current standard of care. Therefore this design would not continue in order to show that the experimental therapy is significantly worse than control.

### Interim monitoring

Interim monitoring for early efficacy or futility begins when 500 patients are enrolled and interim analyses are planned after every additional 100 patient are enrolled for a total of nine possible interim analyses. Interim monitoring for futility is based on the predictive probability that the trial will be successful at the maximum sample size of 1,400 patients, *P*_*max*_. If, at any interim analysis *P*_*max*_ is less than 5%_,_ the trial will stop for futility. Interim monitoring for efficacy is based on the predictive probability that the trial will be successful at the current sample size, *n*, if accrual to the trial would stop and all currently enrolled patients completed follow-up, *P*_*n*_. If, at any interim analysis *P*_*n*_ is greater than 99%, accrual will stop for predicted success. If the trial is not stopped early for futility or if accrual is not stopped early for predicted success (*P*_*max*_ >5% and *P*_*n*_ <99%), then the trial will continue enrollment to the maximum sample size. If accrual stops early for predicted success, or the trial continues to the maximum sample size of 1,400 patients, the primary efficacy analysis will be conducted when all enrolled patients have completed their 90-day follow-up.

### Primary efficacy analysis

The primary efficacy analysis will be conducted after all enrolled patients have completed follow-up for the primary endpoint. We assume the probability of success, *θ*_*j*_, has a Beta prior distribution

$$ \left[{\theta}_j\right]\sim Beta\left(1,\ 1\right), $$

where *j* = C is the control arm and *j* = E is the experimental arm. This prior distribution is equivalent to a uniform prior across the unknown event rates and equates to observing two patients’ worth of information where one experienced a success and one did not. Therefore even with the minimum sample size of 250 patients per group, the prior contributes less than 1% of the information in the posterior. At each interim analysis and at the final analysis the number of observed successes, *x*_*j*_, among the currently enrolled patients, *n*_*j*_, is modeled as a binomial distribution

$$ \left[{x}_j\right]\sim Binomial\left({n}_j,{\theta}_j\right). $$

We update the prior distribution with the currently observed data (*x*_*j*_, *n*_*j*_) and the resulting posterior distribution is

$$ \left[{\theta}_j\Big|{x}_j,{n}_j\right]\sim Beta\left(1+{x}_{j,}1+{n}_j-{x}_j\right). $$

Given the number of successes in each group *x*_*C*_ and x_*E*_ and the total number randomized to each group, *n*_*C*_ and *n*_*E*_, the primary analysis is

$$ \Pr \left({\theta}_E>{\theta}_C\Big|\mathrm{Trial}\ \mathrm{Data}\right)={\displaystyle {\int}_0^1{\displaystyle {\int}_0^{\theta_E}\frac{\Gamma \left(2+{n}_E\right)}{\Gamma \left(1+{x}_E\right)\Gamma \left(1+{n}_E-{x}_E\right)}{\theta}_E^{x_E}{\left(1-{\theta}_E\right)}^{n_E-{x}_E}\frac{\Gamma \left(2+{n}_C\right)}{\Gamma \left(1+{x}_C\right)\varGamma \left(1+{n}_C-{x}_C\right)}}}{\theta}_C^{x_C}{\left(1-{\theta}_C\right)}^{n_C-{x}_C}d{\theta}_Cd{\theta}_E. $$

This value is compared to 0.979 in the final analysis. If the posterior probability that the success rate on the experimental arm, *θ*_*E*_, is greater than the success rate on the control arm, *θ*_*C*_, is greater than 0.979, the treatment will be considered successful,

$$ \Pr \left({\uptheta}_{\mathrm{E}}>{\uptheta}_{\mathrm{C}}\Big|\ \mathrm{Trial}\ \mathrm{Data}\right) > 0.979. $$

The probability of 0.979 is similar to a one-sided critical value of 0.021 in a frequentist trial. This critical value for the final analysis was selected through simulation to control the one-sided Type I error rate of this trial, given the multiple interim analyses, to less than 0.025. SHINE’s two-sided 0.05 and the Goldilocks’ one-sided 0.025 Type I error rates are equivalent. However, the overall Type I error was assessed for the Goldilocks design by simulation. The null space is defined both by the accrual rate and the response rates for both groups. Type I error control was shown by simulation only considering an accrual rate of 33 patients per month and for a response rate of 25% in both arms. The overall Type I error of the design may be larger or smaller at other points in the null space.

### Longitudinal model and predictive probabilities

As described above, interim analyses for efficacy and futility are based on predictive probability calculations. Because of a lag between enrollment and when the primary endpoint is observed, at each interim analysis there will be patients who have complete information through 90 days, more recently enrolled patients who may have only 6-week assessment of mRS, and the most recently enrolled patients who provide only baseline information. For the predictive probability calculations, we utilize the information from patients with incomplete information to the extent that the baseline and 6-week assessments are associated with the primary endpoint at 90 days. Patients with complete information inform this association.

A Bayesian model is built to learn the associations between the earlier 6-week time point and the primary endpoint at 90 days. For each arm, we use three beta-binomial distributions to model the transition:

1. from baseline to 90-days for patients with baseline information only,
2. from 6 weeks to 90-days for patients who were a success at 6 weeks, and
3. from 6 weeks to 90-days for patients who were not a success at 6 weeks.

Because the arms are modeled independently and identically, we present the longitudinal model generically without reference to treatment group. At each interim analysis with a total of *n* patients enrolled, the number of patients with complete follow-up through 90-days is *n*_*c*_. The number of these patients who have achieved a success is *x* and the number of these patients who did not achieve success is *z*. The number of patients enrolled but who have incomplete information is n^*^. Thus *n = n*_*c*_ + n^*^ *= x + z* + n^*^.

For the n^*^ patients with incomplete information, they either 1) have no 6-week follow-up, n^*^_0_, 2) have achieved success at 6 weeks, n^*^_+_ or 3) have not achieved success at 6 week, n^*^_-._ For each of these three groups, we use a beta-binomial model to predict the number of these patients who will be a success on the primary 90-day endpoint. Given the currently observed *n*_*c*_ patients with complete data, the number of patients in each of the three incomplete information groups who will be a success on the primary endpoint is

$$ \begin{array}{l}{x^{*}}_0\Big|\ \mathrm{Current}\ \mathrm{Data} \sim \mathrm{Beta}-\mathrm{binomial}\left({n^{*}}_0,\ 1 + x,\ 1 + z\right)\\ {}{x^{*}}_{+}\Big|\ \mathrm{Current}\ \mathrm{Data} \sim \mathrm{Beta}-\mathrm{binomial}\left({n^{*}}_{+},\ 1 + {x}_{+},\ 1 + {z}_{+}\right)\\ {}{x^{*}}_{-}\Big|\ \mathrm{Current}\ \mathrm{Data} \sim \mathrm{Beta}-\mathrm{binomial}\left({n^{*}}_{-},\ 1 + {\mathrm{x}}_{-},\ 1 + {z}_{-}\right)\end{array} $$

where x and z are the number of patients who are 90-day successes and failures, respectively; *x*_*+*_ and *z*_*+*_ are the number of patients who were 6-week successes who were successes (*x*_*+*_) and failures (*z*_*+*_) at 90-days, respectively; and *x*_*−*_ and *z*_*−*_ are the number of patients who were not 6-week successes who were successes (*x*_*−*_) and failures (*z*_*−*_) at 90-days.

For each value of x^*^_0_, x^*^_+_, x^*^_−_*,* there is an associated probability based upon the described distributions, and correspondingly, there is an associated probability for every possible number of total successes if all patients were to complete follow-up.

For each pair of possible total successes between the experimental and control group, we can determine if that combination would result in a success on the primary efficacy analysis, whether Pr(*θ*_*E*_ > *θ*_*C*_ >0.979). Summing the probabilities for the cases that would result in trial success is the predictive probability of trial success for the currently enrolled patients.

For the predictive probability of success at the maximum sample size, we perform a similar calculation but assume the trial continues enrollment to the maximum sample size of 1,400 patients. However, this requires calculation of the predictive distribution of success for patients not yet enrolled. These future patients are included in the predictive probability calculation described above as patients that have only a baseline assessment (no available interim data).

## Results

Table 2 summarizes the differences between the two designs. Both designs are innovative and complex, and both offer many features that help address the primary objective of the trial. There is little common ground for a head-to-head comparison, except to explore how each design behaves in terms of power and mean sample size across a range of scenarios.

**Table 2:** **Summary of design features for SHINE and the Goldilocks alternative**

	**SHINE**	**Goldilocks**
Hypothesis testing	Two-sided	One sided
Primary analysis	Covariate adjusted	Unadjusted
Randomization	Response adaptive	1:1
Minimum sample size	Approx 600	Fixed at 500
Maximum sample size	Between 1,400 and 1,718	Fixed at 1,400
Planned interim analyses	4	9
Stopping boundaries	Group sequential	Predictive probabilities, incorporating early information
Complete follow-up of all patients in event of early termination	Undefined critical value	Explicitly defined critical value

To determine the operating characteristics of each design, we simulated both the SHINE design and the Goldilocks design across a range of treatment effects and a range of success rates for the control group. The SHINE design was simulated including the group sequential stopping boundaries, the sample size re-estimation, and using a chi-square test between treatment groups as the primary analysis (consistent with the primary power calculation). The response adaptive randomization component of the design was not included in order to preserve the operational integrity of the trial. The Goldilocks design was simulated exactly as described above. Simulation code was written in the R statistical language [10] and 10,000 trials were simulated for each scenario. All simulations assume no lost to follow-up, and an accrual rate of 33 patients per month. Thus, at each interim analysis there are approximately 100 patients enrolled but without complete follow-up through 90 days. The nature of the longitudinal data necessary for simulation of the Goldilocks design was estimated based on the transitions in mRS observed within the raw data from the NINDS tPA trial including both the tPA and control groups since SHINE includes both [11].

### Sample size

**Figure 1:**
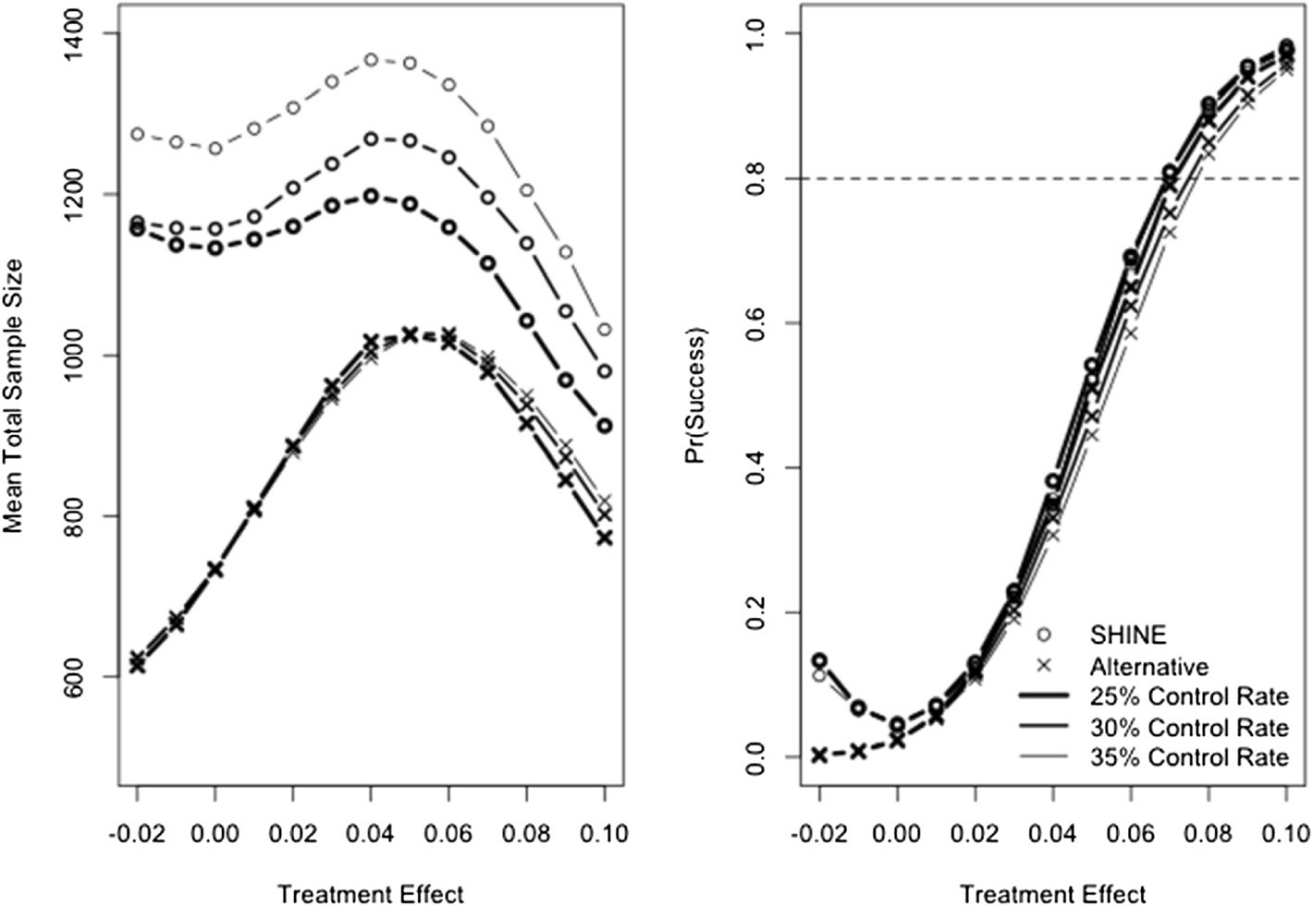
**Mean total sample size enrolled (left panel) and probability of trial success (right panel).** SHINE is plotted with a circle and the Goldilocks is plotted with an x. Heavy line represents a control success rate of 25%, medium line represents a control success rate of 30%, and light line represents a control rate of 35%. Dashed line on the right panel shows 80% power for reference.

The left panel of Figure 1 shows the total mean number of patients enrolled for the two designs. Generally, when the treatment effects are null or small, both designs stop early for futility and when the treatment effects are large, both designs stop early for success. Thus, sample sizes are smaller at each end of the treatment effect range than in the middle.

Across all simulated scenarios, the SHINE design tends to enroll a greater number of patients than the Goldilocks design. The difference in sample size between the two designs is attributable to a different number of interim analyses, different mechanisms for early stopping, differing aggressiveness of the stopping boundaries, and that SHINE is based on a two-sided alternative hypothesis while the Goldilocks design is based on a one-sided alternative hypothesis.

The SHINE design includes four interim analyses and the Goldilocks design includes nine. With more interim analyses, the Goldilocks design has more opportunities to stop early. Additionally, the two designs have different mechanisms for early stopping and differently account for complete follow-up of all enrolled patients should the trial stop early. Early stopping for the SHINE design is based on the information fraction, or the number of patients with observed 90-day outcomes. It stops early only when observed 90-day data produces an overwhelming effect for efficacy or futility. For example, when 1,000 patients are enrolled, we expect that 900 patients will have complete data. The trial will stop for efficacy if, based on these 900 patients, the *P* value is less than 0.008 (Table 1) and the trial is considered a success. There is no defined critical value for a later analysis that might include the 100 outstanding patients once they have completed follow-up.

Early stopping in the Goldilocks design is based on the number of patients enrolled and allows for complete follow-up of all enrolled patients. Thus, the Goldilocks design is able to have more aggressive early stopping behavior, stopping accrual and then allowing for the additional follow-up of enrolled patients in order to observe the necessary information for success, whereas the SHINE design stops only once the necessary information for success is already observed. The Goldilocks design might stop when 900 patients are enrolled, and 800 patients have complete data, as long as there is a high probability that including the outstanding 100 patients in the final analysis (many of whom have interim 6-week mRS values) would result in trial success. In the Goldilocks design, all enrolled patients are explicitly included in the final analysis.

The difference in expected sample size is largest when the control is only slightly better than the experimental arm. The alternative hypothesis for the SHINE design is two-sided, but is one-sided for the Goldilocks design. This contributes to different stopping behavior when the control arm is only slightly better than the treatment. In these scenarios, the Goldilocks design stops early for futility, reacting to the fact that it is unlikely the experimental treatment will be proven to be superior to the control. The SHINE design continues in these scenarios, not crossing a futility boundary (the treatments are the same), and not seeing a large enough treatment effect to cross a success boundary (the control is significantly better than the experimental).

### Probability of trial success

The right panel of Figure 1 shows the probability of trial success for the two designs. When the treatment effect is null, SHINE has an analytically controlled two-sided overall Type I error rate of 5% and the Goldilocks design has a simulated one-sided Type I error rate of approximately 2.5%. We present only one null scenario, and the Type I error rate for the Goldilocks design may vary across the null space. When the treatment arm is slightly worse than control, the SHINE design may conclude success of control over treatment, whereas the Goldilocks design cannot. Thus, the SHINE design has a higher probability of trial success in these scenarios.

When the control rate is 25%, the probability of success in scenarios where the treatment is better than control is similar between the two designs. As the control rate increases, a small amount of power is lost in the Goldilocks design. The Goldilocks design loses approximately 2% power when the control rate increases from 25% to 30%, and approximately another 2% power when the control rate increases from 30% to 35%.

**Figure 2:**
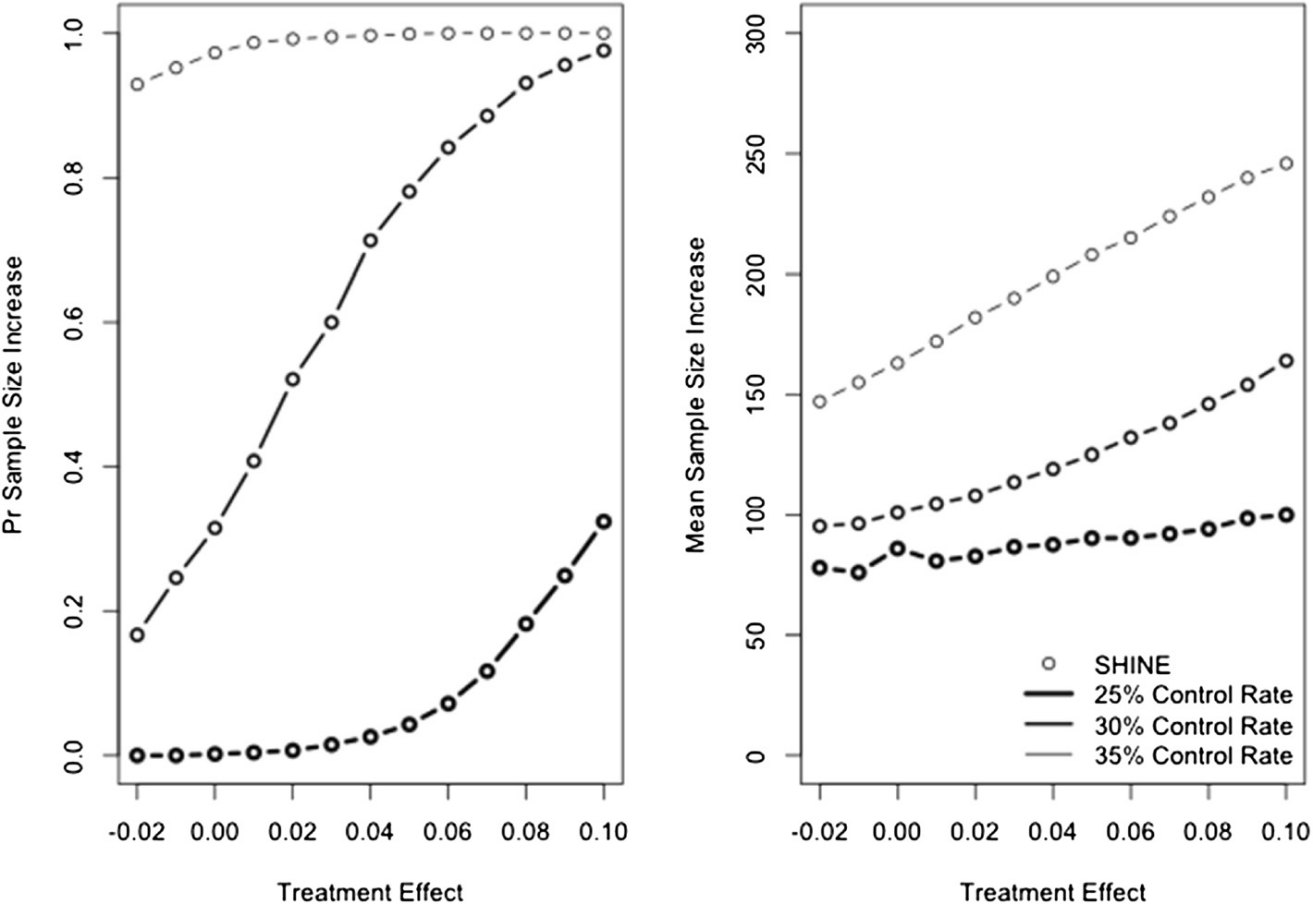
**Probability of increasing the maximum sample size (left panel) and mean number of patients added (right panel).** SHINE is plotted with a circle (Goldilocks design does not include sample size re-estimation). Heavy line represents a control success rate of 25%, medium line represents a control success rate of 30%, and light line represents a control rate of 35%.

The SHINE design includes a sample size re-estimation to preserve power if the control rate is higher than expected. Thus, as the control rate increases, the power of the design is unchanged. However, the mean sample size increases (left panel Figure 1). In the most extreme case, the Data Safety and Monitoring Board (DSMB) could be asked to increase the sample size from 1,400 to 1,718 patients. Figure 2 shows the probability that the SHINE maximum sample size is increased and the mean number of patients added. The size of the sample size re-estimation is proportional to the pooled success rate and so depends on both the control success rate and the treatment effect. Because the sample size re-estimation is blinded it is not possible to diagnose whether a high pooled event rate is due to a higher than expected control rate, or to a larger than expected treatment effect. In fact, because the treatment effect is fixed at 7% in the sample size re-estimation procedure, a high pooled event rate is always attributed to a higher than expected control rate. As a result, even when the true control rate is 25%, a large treatment effect can trigger a sample size increase and the larger the treatment effect, the larger the increase to the maximum sample size. However, with a large treatment effect, the trial is then also likely to stop early for success. The trial’s mean sample size does not increase by exactly the number of patients added to the maximum sample size because of this potential for early success stopping. However, the potential delay to the first interim look may translate to an increase in the mean sample size.

### Compare and contrast

The strengths of the SHINE design include the sample size re-estimation and the covariate adjusted final analysis. SHINE has analytical control of overall Type I error, and as simulated, the SHINE design offers a slight advantage in terms of power. Covariates were not included in the simulation study, but a covariate adjusted primary analysis is likely to increase the trial’s overall power from what was simulated. With fewer interim analyses, there is also less operational complexity. However, the adaptive randomization component was not included in the simulations. This type of randomization scheme has the potential to increase operational complexity and reduce power. While response adaptive randomization would likely randomize more patients to the more effective therapy, the power of the trial could be reduced because in a two-arm trial, equal allocation between arms should provide maximum power.

The strengths of the Goldilocks design include more frequent interim analyses, the longitudinal model of the primary outcome, and explicitly accounting for patients with incomplete follow-up if accrual to the trial where to stop early. The Goldilocks design offers advantages in terms of sample size. If accrual to the trial is faster than expected, the difference in sample size between the two designs will be greater than described here. If accrual to the trial is slower than expected, the difference in sample size will be smaller. Accrual to clinical trials is frequently slower than expected. While the Goldilocks design randomizes patients equally to the two treatment arms, an increased potential to stop the trial earlier would allow results to be communicated earlier and would allow patients outside the trial to be treated with the more effective therapy sooner.

The comparison of operating characteristics should be interpreted with caution bearing in mind the many differences between the two designs (Table 2), in particular the differences in the stopping boundaries and the limitations of the simulation study, specifically that covariates and the adaptive randomization component of the SHINE design were not included. The operating characteristics of the Goldilocks design, in particular Type I error control, are assessed with simulation and so the Type I error rates of the two designs may not be exactly equal across the entire null space. While we see that the SHINE design offers advantages in terms of power and that the Goldilocks offers advantages in terms of sample size, we expect that a greater sample size would confer greater power. The largest difference in power we observed in the simulations was when the control rate was 35% and the treatment rate was 41%. In this scenario SHINE has 9.5% more power (58.6% versus 68.1%) and enrolls an average of 308 additional patients.

None of the simulated scenarios showed a similar sample size between the two designs, the Goldilocks design always had a smaller mean sample size. In the primary expected scenario of 32% vs. 25%, both designs are tuned to have approximately 80% power, and the average sample size in SHINE is 135 patients higher than in the Goldilocks design (1,114 vs. 979). For similar powers the Goldilocks trial saves at least 100 patients, oftentimes more. When the difference between arms is very small, only 2%, the two designs have similar power, approximately 12%, regardless of the control rate. The SHINE design averages 273, 322, and 429 more patients at control rates of 25%, 30%, and 35%, respectively. When the treatment effect is large, 10%, SHINE has an additional 1 to 3% power, but averages and additional 139, 178, and 213 patients at control rates of 25%, 30%, and 35%, respectively.

### Trial execution

SHINE is currently ongoing. The Goldilocks design will be virtually executed upon completion of SHINE using the observed patient data from SHINE. The purpose of this virtual trial execution is to determine the resources used and resulting evidence for the comparison of the experimental versus control arms had SHINE been conducted according to the Goldilocks design. The outcomes of the two trial executions can be compared in terms of final trial conclusions, total number of patients enrolled, number of patients enrolled to the most effective treatment arm, and trial duration.

This exercise will require datasets that provide snapshots of the accumulated data from SHINE to conduct each of the planned interim analyses and the final analysis. The trial’s data coordinating center has agreed to provide these snap shots and store these blinded datasets until the SHINE results are released. If SHINE stops accrual early, but the Goldilocks design indicates continuing enrollment, patients for the future interim analyses for the Goldilocks design will be simulated by re-sampling the observed SHINE patients. We emphasize that we have a prospectively defined design and execution plan. Limitations in the re-execution approach will be assessed and reported at the time the re-execution is performed.

## Discussion

The original SHINE design presented within ADAPT-IT included response adaptive randomization and had one futility analysis (700 patients with complete data, 50% information) and one early success stopping analysis (938 patients with complete data, 67% information). As the work within ADAPT-IT progressed, the potential benefits of additional interim analyses were discussed. The number of interim analyses for SHINE was re-evaluated and the design was updated to the four interim analyses for early stopping as described here. The Goldilocks design was then finalized. External to the ADAPT-IT process, a sample size re-estimation was then added to the SHINE design and the simulation exercise presented here was then performed. Certainly, the two designs could continue to be iterated to be more similar or pieces of one design could be included in the other. For example, the Goldilocks design could allow a larger sample size and a covariate adjusted final analysis, which are strengths in the SHINE trial design. The SHINE design could have more frequent interim analyses or account for patients with incomplete follow-up when the study stops, which are strengths in the Goldilocks design. However, we present the Goldilocks design as it was developed at the time and we present the SHINE design as it is being conducted.

The SHINE investigators were tasked with choosing only a single design to implement and ultimately elected not to implement the Goldilocks alternative. A main aspect for consideration was the potential for formal ‘flip-flop’. The Goldilocks design stops accrual for predicted success if the probability for success with the currently enrolled patients, allowing for the outstanding (approximately) 100 patients to reach their 90-day endpoint, is greater than 99%. There is a small chance that the trial could stop for predicted success, complete follow-up of the enrolled patients, and then at the final analysis, miss the 0.979 posterior probability threshold required for trial success. For example, in the scenario where the experimental treatment offers a 7% absolute benefit, the trial stopped early for predicted success 62% of the time and in 0.5% of those cases, failed to obtain the final critical value for success. Most likely, if such a flip-flop occurs, the experimental treatment is not effective. While such flip-flops occur in 0.5% of early stopping cases when the treatment is effective (+7%) they occurred in 6% of cases when the treatment is ineffective - largely because of regression to the mean in those cases and this in fact helps to conserve Type I error. Even if this were to occur, the final *P* value would likely still be just a little larger than the critical value.

It is important to recognize that this potential exists in a group sequential design as well. While such an occurrence is similarly unlikely, it is not well characterized or formally accounted for. Stopping based on predictive probabilities explicitly accounts for complete follow-up of all enrolled patients and so it is natural to quantify the ‘flip-flop’. Group sequential stopping is typically based on the amount of information observed and while methods exist to account for ‘overrun’ [12], the typical implementation of group sequential designs does not dictate how to handle patients with incomplete information should the trial stop early. These designs typically do not include a formal analysis on all enrolled patients and so do not specify the critical value needed for such an analysis. Our simulations of the SHINE design show that in this case such an analysis would likely not meet the *P* value that was required for early stopping, simply due to regression to the mean, but is likely to meet the *P* value required for success at the end of the trial with complete information (data not shown).

A second consideration for the SHINE investigators surrounding the Goldilocks design was a wholesale change of the design after it had already been through the NIH peer review process and had been selected for funding. Additionally, there was concern from the SHINE PI about whether the design and final analysis would be accepted by the clinical community given the small number phase III trials that have utilized a Bayesian approach.

One of the larger differences between the two designs is that SHINE is a two-sided alternative hypothesis while the Goldilocks design is one-sided. Although the error rates are similar, differences in sample size and power between the two designs are confounded with this difference in hypothesis testing in the setting where the standard of care arm is slightly better than the tight control arm. While two-sided trials are standard, and hence the choice for the SHINE investigators, the Goldilocks design considers it unlikely that a DSMB would let a trial proceed only to show that an expensive, more laborious therapy is in fact significantly inferior to the standard of care. However, the clinical community is currently quite divided on the utility of intensive glucose control in stroke and both the SHINE and the NETT leadership believe that the greatest opportunity for SHINE to leave stroke physicians with a definitive answer is through the two-sided design. Certainly, there are many stroke physicians who would be satisfied with the one-sided hypothesis tested by the Goldilocks design, yet there is a concern that practice will not be changed unless there is a definitive answer in favor of one treatment or the other. Of course, even the two-sided design may end in the null space; given the additional resources required to perform the intense glucose controls it is likely that most would abandon this treatment.

The primary lesson learned in the ADAPT-IT design process for SHINE was about the value of trial simulation. Trial simulation may typically be associated Bayesian designs or with more complex adaptive designs, such as where a close form solution for Type I error and power are not available. In the ADAPT-IT design process, clinical trial simulation illuminated several points of discussion with both designs. Clinical trial simulation of the Goldilocks design was necessary to understand its early stopping behavior and the potential for ‘flip-flop’. A remedy for ‘flip-flop’ is to increase the threshold required for early stopping, which was done during the design process, but this will decrease the probability of early stopping and increase mean sample size. Such trade-offs can only be assessed through the process of clinical trial simulation. Trial simulation also illuminated the differences in trial behavior due to two-sided versus one-sided hypothesis testing for the investigators and initiated the discussion about which test would ultimately be used in the conduct of the trial.

Finally, the SHINE design includes two ‘well understood’ [13] adaptive trial features that are commonly used in phase III trials: a blinded sample size re-estimation and group sequential stopping boundaries. While the statistical properties of each are completely understood separately, combining two well-understood design features does not add up to a well-understood design. For example, when the experimental therapy is performing better than control, the sample size may be increased due to the sample size re-estimation and the first interim analysis will be delayed. The trial may stop for success, though with additional patients and more time than it might require otherwise. Given that the experimental therapy is performing well, the additional patients and time could be undesirable. On the other hand, the delay could be considered small and worth the protection of power that the sample size re-estimation provides. Even with the simulation exercise presented here, the SHINE design is still not completely characterized because covariates and the adaptive randomization component are not included.

## Conclusions

The goal of this project was to illustrate two alternative real-world designs, the two primary designs considered for a multi-center NIH-funded randomized clinical trial. Because they are two real-world designs, simple head-to-head comparisons changing one feature at a time are not possible and the alternative trials must be judged on the totality of their operating characteristics.

The consideration of alternative trial designs was a successful venture in that two designs were brought forward, and both were evaluated, revised, and improved based on the input of all parties involved in the ADAPT-IT process. Simulation of an entire design as it will be conducted was necessary to evaluate the selected design features in concert with each other and to inform the study team of the gains/losses associated with various design choices. Trial simulation should not be reserved for only complex adaptive designs. Rather, all trialists should consider trial simulation an important tool for developing a complete understanding of the statistical properties of their trial design [14].

## Appendix

The following list contains the ethics boards or institutional review boards that approved the SHINE study at each participating clinical site in the Neurological Emergency Treatment Trials Network (Table 3).

**Table 3:** **Ethical body at each enrolling site**

**Spoke**	**IRB name**
University of Arizona Medical Center - University Campus, Tucson, AZ	University of Arizona Human Subjects Protection Program IRB
University of Arizona Medical Center - South Campus, Tucson, AZ	University of Arizona Human Subjects Protection Program IRB
University of Cincinnati Medical Center, Cincinnati, OH	University of Cincinnati Institutional Review Board
Emory University Hospital, Atlanta, GA	Emory University Institutional Review Board
Emory University Hospital Midtown, Atlanta, GA	Emory University Institutional Review Board
Grady Memorial Hospital, Atlanta, GA	Emory University Institutional Review Board
University of Michigan Medical Center, Ann Arbor, MI	University of Michigan IRBMED
Henry Ford Hospital, Detroit, MI	Henry Ford Health System Institutional Review Board
University of Kentucky Hospital, Lexington, KY	University of Kentucky Medical Institutional Review Board
University of Maryland Medical Center, Baltimore, MD	University of Maryland, Baltimore (UMB) Institutional Review Board (IRB)
Massachusetts General Hospital	Partners Human Research Committee
Hennepin County Medical Center, Minneapolis, MN	Hennepin County Medical Center Human Subjects Research Review Committee
University of Kansas Hospital, Kansas City, KS	Kansas University Medical Center Institutional Review Board
University of Minnesota Medical Center Fairview, Minneapolis, MN	University of Minnesota Institutional Review Board
NYP Columbia University Medical Center, New York, NY	Columbia University Medical Center Institutional Review Board
OSU Wexner Medical Center, Columbus, OH	Biomedical Sciences Institutional Review Board
Summa Akron City Hospital, Akron, OH	Summa Health System Institutional Review Board
Harborview Medical Center, Seattle, WA	University of Washington Human Subjects Division Institutional Review Board
UPMC Presbyterian Hospital, Pittsburgh, PA	University of Pittsburgh Institutional Review Board
UPMC Mercy Hospital, Pittsburgh, PA	University of Pittsburgh Institutional Review Board
NYP Weill Cornell Medical Center, New York, NY	Weill Cornell Medical College Institutional Review Board
WVU Healthcare Ruby Memorial Hospital, Morgantown, WV	West Virginia University Institutional Review Board (IRB)
Buffalo General Medical Center, Buffalo, NY	SUNY University at Buffalo Health Sciences IRB (HSIRB)
Northwestern University	Northwestern University Biomedical IRB
UT Southwestern - Parkland Hospital	UT Southwestern Institutional Review Board (IRB)
UT Southwestern University Hospital-Zale Lipshy, Dallas, TX	UT Southwestern Institutional Review Board (IRB)
Baylor College of Medicine, Houston, TX	Baylor College of Medicine Institutional Review Board
Vanderbilt University Medical Center	Vanderbilt University Institutional Review Board
St. Thomas Hospital, Nashville, TN	Sterling Institutional Review Board
UVA Medical Center, Charlottesville, VA	University of Virginia Institutional Review Board for Health Sciences Research
OSF Saint Francis Medical Center	University of Illinois College of Medicine at Peoria Institutional Review Board
Penn State Hershey Medical Center, Hershey, PA	Penn State Hershey College of Medicine Institutional Review Board
Georgia Regents Medical Center, Augusta, GA	Georgia Regents University Institutional Review Board
University of Utah Healthcare, Salt Lake City, UT	University of Utah Institutional Review Board
Mayo Clinic, Jacksonville, FL	Mayo Clinic Institutional Review Board
Stanford University Medical Center, Stanford, CA	Stanford University Human Subjects Committee IRB
SUNY Downstate	Biomedical Research Alliance of New York (BRANY) Institutional Review Board
Kings County Hospital	Biomedical Research Alliance of New York (BRANY) Institutional Review Board
Lincoln Medical and Mental Health Center, Bronx, NY	Biomedical Research Alliance of New York (BRANY) Institutional Review Board
Maimonides Medical Center	Biomedical Research Alliance of New York (BRANY) Institutional Review Board
Allegheny General Hospital	Allegheny Singer Research Institute Institutional Review Board
Thomas Jefferson University Hospital, Philadelphia, PA	Thomas Jefferson University Institutional Review Board
Hackensack University Medical Center, Hackensack, NJ	Hackensack UMC Institutional Review Board
Temple University Hospital, Philadelphia, PA	Temple University Institutional Review Board
University Medical Center Brackenridge, Austin, TX	Seton Institutional Review Board
Seton Medical Center, Austin, TX	Seton Institutional Review Board
Valley Baptist Medical Center - Harlingen, Harlingen, TX	Valley Baptist Medical Center Institutional Review Board
Memorial Hermann Texas Medical Center, Houston, TX	UT Health Committee for the Protection of Human Subjects
Long Beach Memorial Medical Center, Long Beach, CA	Memorialcare Health System Institutional Review Board
Ronald Reagan UCLA Medical Center, Los Angeles, CA	University of California Los Angeles Institutional Review Board
San Francisco General Hospital, San Francisco, CA	University of California at San Francisco Committee on Human Research
UCSF Medical Center, San Francisco, CA	University of California at San Francisco Committee on Human Research
California Pacific Medical Center Pacific Campus, San Francisco, CA	Sutter Health Institutional Review Board
California Pacific Medical Center Davies Campus, San Francisco, CA	Sutter Health Institutional Review Board
Hospital of the University of Pennsylvania, Philadelphia, PA	University of Pennsylvania Institutional Review Board
WellSpan York Hospital, York, PA	WellSpan Health Institutional Review Board
Abington Memorial Hospital, Abington, PA	Abington Memorial Hospital Institutional Review Board
VCU Medical Center, Richmond, VA	Virginia Commonwealth University Institutional Review Board
Detroit Receiving Hospital, Detroit, MI	Wayne State University Institutional Review Board
Sinai-Grace Hospital, Detroit, MI	Wayne State University Institutional Review Board
William Beaumont Hospital-Troy, Troy, MI	Beaumont Research Institute Human Investigation Committee
William Beaumont Hospital, Royal Oak, MI	Beaumont Research Institute Human Investigation Committee
Froedtert Memorial Lutheran Hospital, Milwaukee, WI	Medical College of Wisconsin Institutional Review Board

## Abbreviations

ADAPT-IT: Adaptive Designs Accelerating Promising Trials into Treatments
DSMB: Data safety and monitoring board
FDA: Food and Drug Administration
IV: Intravenous
IRB: Institutional review board
mRS: Modified Rankin Score
NETT: Neurologic emergencies treatment trials
NIH: National Institutes of Health
NIHSS: NIH Stroke Scale
NINDS: National Institute of Neurologic Disorders and Stroke
SHINE: The Stroke Hyperglycemia Insulin Network Effort
